# Focus on long non-coding RNA MALAT1: Insights into acute and chronic lung diseases

**DOI:** 10.3389/fgene.2022.1003964

**Published:** 2022-09-16

**Authors:** Xingning Lai, Jie Zhong, Aihua Zhang, Boyi Zhang, Tao Zhu, Ren Liao

**Affiliations:** ^1^ Department of Anesthesiology, West China Hospital, Sichuan University, Chengdou, Sichuan, China; ^2^ Research Unit for Perioperative Stress Assessment and Clinical Decision, Chinese Academy of Medical Sciences (2018RU012), West China Hospital, Sichuan University, Chengdou, Sichuan, China

**Keywords:** MALAT1, acute lung injury, chronic lung disease, miRNA, inflammation

## Abstract

Acute lung injury (ALI) is a pulmonary illness with a high burden of morbidity and mortality around the world. Chronic lung diseases also represent life-threatening situations. Metastasis-associated lung adenocarcinoma transcript 1 (MALAT1) is a type of long non-coding RNA (lncRNA) and is highly abundant in lung tissues. MALAT1 can function as a competitive endogenous RNA (ceRNA) to impair the microRNA (miRNA) inhibition on targeted messenger RNAs (mRNAs). In this review, we summarized that MALAT1 mainly participates in pulmonary cell biology and lung inflammation. Therefore, MALAT1 can positively or negatively regulate ALI and chronic lung diseases (e.g., chronic obstructive pulmonary disease (COPD), bronchopulmonary dysplasia (BPD), pulmonary fibrosis, asthma, and pulmonary hypertension (PH)). Besides, we also found a MALAT1-miRNA-mRNA ceRNA regulatory network in acute and chronic lung diseases. Through this review, we hope to cast light on the regulatory mechanisms of MALAT1 in ALI and chronic lung disease and provide a promising approach for lung disease treatment.

## Introduction

Acute lung injury (ALI) is considered to be an important cause of high morbidity and mortality worldwide and occurs due to damage to the pulmonary system (Vishnupriya et al., 2020). ALI is a severe respiratory syndrome with the existence of hypoxemia. Acute respiratory distress syndrome (ARDS) represents a serious form of ALI. ALI and ARDS are common among critically ill patients (Mokra et al., 2019). There are many risk factors related to ALI, including direct (pulmonary) injury (e.g., pneumonia, gastric aspiration, pulmonary contusion, alveolar hemorrhage, etc.) and indirect (extrapulmonary) injury (e.g., severe sepsis, transfusions, pancreatitis, and shock) (Ware and Matthay, 2000). Although treatments have been updated, there are no specific therapeutic strategies for ALI and ARDS. Many ALI or ARDS patients need positive-pressure ventilation to maintain adequate oxygenation. One study found that some therapies, like corticosteroids, extracorporeal support, fluid management, and vasodilators, have the potential to treat ALI (Wheeler and Bernard, 2007). Accumulative evidence indicates that chronic lung diseases also remain the leading cause of death and disability around the world. The prevalence of chronic respiratory diseases is associated with age, sex, income, and region (2020). Chronic pulmonary diseases include chronic obstructive pulmonary disease (COPD), bronchopulmonary dysplasia (BPD), pulmonary fibrosis, asthma, and pulmonary hypertension (PH) (Worthington and Hagood, 2020; Kwok et al., 2021). Effective treatments for chronic lung diseases are urgently needed these days (Riley and Sciurba, 2019).

Long non-coding RNAs (lncRNAs) (≥200 nucleotides in length) and microRNAs (miRNAs) (∼21 nucleotides in length) are small non-coding RNAs without the ability to encode proteins (Beermann et al., 2016; Chen L. et al., 2019). These two non-coding RNAs modulate gene transcription and translation by interacting with the 3′ untranslated region (3′UTR) of messenger RNA (mRNA) (Krol et al., 2010; Dykes and Emanueli, 2017). Functionally, lncRNAs and miRNAs modulate various cellular processes, such as cell proliferation, differentiation, and apoptosis (Lee and Dutta, 2009; Shi et al., 2013). LncRNA metastasis-associated lung adenocarcinoma transcript 1 (MALAT1, also called nuclear enriched abundant transcript 2 (NEAT2) was first found in non-small cell lung cancer (NSCLC) (Ji et al., 2003). The MALAT1 encoding gene is located on mouse chromosome 19qA and human chromosome 11q13.1 (Zhang et al., 2012). The primary MALAT1 transcript is about 8 kb in humans and 6.7 kb in mice (Sun and Ma, 2019). MALAT1 influences various physiopathological processes, including tissue inflammation, embryo implantation, angiogenesis, cardiovascular remodeling, and tumor progression (Lei et al., 2018). For example, MALAT1 exhibits inhibitory and positive effects on cancer metastasis (Huang et al., 2019). MALAT1 also acts as a competitive endogenous RNA (ceRNA) to sponge miRNAs and reduce miRNA activity, finally, impairing the interaction between miRNAs and their targeted genes (Su et al., 2021). For instance, MALAT1 regulates lung inflammation *via* sponging miRNAs in lung injury (Chen et al., 2021). Previous studies indicated that MALAT1 can be identified as a prognostic or diagnostic marker in human diseases (Goyal et al., 2021). And accumulative evidence confirmed that targeting MALAT1 might be a potential therapeutic way to alleviate tissue injury (Abdulle et al., 2019).

In this review, we collected the evidence to discuss MALAT1 effects on ALI and chronic lung diseases, altogether describing the roles of MALAT1 in inflammation responses, cell apoptosis, cell proliferation, and airway remodeling. In addition, there are also targeted therapies for the treatment of lung diseases.

## LncRNA metastasis-associated lung adenocarcinoma transcript 1 and airway microenvironment

The lung is the primary organ for gas exchange and consists of the airways and distal alveoli. The lung is continuously open to the external environment, thereby resulting in high-frequency exposure to air pollution, infectious agents, and toxic antigens (Kwok et al., 2021). The airway modulates lung fluid balance, elimination of inhaled agents, attraction and activation of inflammatory cells, and airway smooth muscle function. In normal conditions, the airway epithelium forms an essential tissue barrier and is the first line of defense against external and internal challenges (Knight and Holgate, 2003; Crystal et al., 2008). In response to toxic stimuli, lung epithelial cell damage destroys the epithelial barrier and leads to airway injury (Shaykhiev et al., 2011). The epithelial damage also activates multiple immune cell types [e.g., macrophages, dendritic cells (DCs), etc.], which contributes to further inflammatory immune responses (Fujita et al., 2015). LncRNA MALAT1 expression is increased in airway epithelial cells (AECs) co-cultured with DCs. When MALAT1 expression is down-regulated in the AECs-DCs co-culture system, the maturity surface marker (CD80, CD86, and major histocompatibility complex class II) expression, inflammatory cytokine [tumor necrosis factor-α (TNF-α), interleukin-6 (IL-6), and interferon γ (IFN-γ)] production, and chemokine [C-X-C receptor 2 (CXCR2) and CXCR4] secretion are all increased in DCs (Li Z. et al., 2018). MALAT1 suppression also reduces cell apoptosis and stimulates cell viability in DCs co-cultured with AECs. These results suggest that MALAT1-regulated crosstalk between AECs and DCs alters the maturation, cytokine production, chemotaxis, and cell apoptosis of DCs (Li Z. et al., 2018).

## LncRNA metastasis-associated lung adenocarcinoma transcript 1 and acute lung injury

ALI is a life-threatening syndrome with acute hypoxemic respiratory failure, bilateral pulmonary infiltrates, and absent left atrial hypertension (Rubenfeld et al., 2005). In ALI, resident lung cells are stimulated to generate chemoattractants, which can recruit inflammatory cells (i.e. neutrophils, and macrophages) into the airway microenvironment. Inflammatory cells produce various proinflammatory cytokines and thus impair the normal pulmonary microenvironment. These inflammatory responses eventually lead to lung injury (Bhatia et al., 2012). Recently, lipopolysaccharide (LPS) induced *in vitro* and *in vivo* models are widely used to study ALI pathogenesis (Xia et al., 2022). MiR-181a-5p expression is decreased in pulmonary arterial hypertension (PAH) rats and its up-regulation decreases right ventricular systolic pressure and lung injury (Zhao et al., 2020). LncRNA MALAT1 expression is enhanced in the plasma of ALI patients. MALAT1 depletion inhibits Fas (apoptotic receptor) via increasing miR-181a-5p (Liu et al., 2021). Suppressing MALAT1 abrogates cell apoptosis and inflammatory responses through the miR-181a-5p-Fas axis in LPS-induced *in vitro* models of ALI. MALAT1 silence and miR-181a-5p over-expression mitigate ALI symptoms (e.g., hypoxemia, and lung edema) *in vivo* (Liu et al., 2021). Chen et al. found that miR-194 expression is declined in lung tissues obtained from LPS-exposed mice. MiR-194 mimic increases miR-194 expression and attenuates lung cell inflammation in response to LPS (Chen R. et al., 2020). One study showed that loss of forkhead box P2 (FOXP2), a transcription factor, accelerates lung alveolarization defects (Shu et al., 2007). MiR-194-5p inhibition can elevate FOXP2 expression. MALAT1 expression can be stimulated by LPS in human pulmonary alveolar epithelial cells (HPAEpiCs) (Nan et al., 2020). MALAT1 suppression increases miR-194-5p to reduce FOXP2, contributing to decreasing cell apoptosis in LPS-treated HPAEpiCs. These results indicate a positive relationship between MALAT1 and LPS-induced ALI (Nan et al., 2020). The transcription factor forkhead box protein A1 (FOXA1) deficiency has been found to inhibit pulmonary epithelial cell apoptosis in ALI (Song et al., 2009). In LPS-treated A549 cells (human alveolar epithelial cell line), miR-17-5p inhibitor increases mRNA and protein expressions of FOXA1. Reducing MALAT1 augments miR-17-5p expression. MALAT1 down-regulation boosts cell proliferation and impedes cell apoptosis through the miR-17-5p-FOXA1 axis in LPS-treated A549 cells (Wei S. et al., 2019). The nuclear factor-kappaB (NF-κB) signaling pathway is proven to participate in mediating inflammation and innate immunity (Hoesel and Schmid, 2013). Myeloid differentiation factor 88 (MyD88) is a target of miR-149. Up-regulated miR-149 inactivates the NF-κB pathway and suppresses MyD88, TNF-α, IL-1β, and IL-6 levels in A549 cells under LPS exposure (Liang et al., 2019). MALAT1 can sponge miR-149. MALAT1 deficiency impairs inflammatory responses by disturbing the miR-149-MyD88-NF-κB signal axis in LPS-induced ALI models (Liang et al., 2019). It is proven that the lung injury score can be used to evaluate the severity of acute lung damage according to histopathological changes (e.g., edema, alveolar inflammation, and interstitial hemorrhage) in lung tissues (Xiao et al., 2021). Dai et al. revealed that MALAT1 acts as an endogenous sponge for miR-146a and abates miR-146a expression. MALAT1 repression blocks LPS-related inflammatory responses *via* sponging miR-146a in murine alveolar macrophages (Dai L. et al., 2018). The injection of small hairpin RNAs (shRNAs) of MALAT1 (sh-MALAT1) significantly decreases MALAT1 expression. The sh-MALAT1 injection abrogates inflammatory factor (IL-6, TNF-α, and IL-1β) production and reduces lung injury score *in vivo*. These results indciate a positive relationship between MALAT1 and acute lung injury (Dai L. et al., 2018). One study discovered that the knockout of MALAT1 decreases the number of neutrophils and macrophages in bronchoalveolar lavage fluid (BALF) samples in ALI mice (Lin L. P. et al., 2019). BML-111, a lipoxin receptor agonist, has been demonstrated to reduce rat acute liver injury (Yan et al., 2016). Li et al. indicated that BML-111 injection relieves ALI symptoms. MALAT1 levels can be increased by BML-111 injection in ALI rats (Li H. et al., 2018). MALAT1 over-expression inhibits the expression of NF-κB and p38 mitogen-activated protein kinase (MAPK) *via* suppressing Toll-like receptor 4 (TLR4), which can eventually reduce LPS-stimulated cell apoptosis and inflammatory factor (IL-6 and TNF-α) production in rat pulmonary microvascular endothelial cells (PMECs) (Li H. et al., 2018). The drying wet ratio of the lung was used to assess the level of pulmonary edema. Silencing MALAT1 increases lung injury score, lung drying wet ratio, and the expression of inflammatory factors IL-6 and TNF-α *in vivo*. These results suggest a negative relationship between MALAT1 and acute lung injury (Li H. et al., 2018).

ARDS is recognized as an acute inflammatory lung injury with excessive pulmonary vascular permeability, increased lung weight, and decreased aerated lung tissue (Ranieri et al., 2012; Bellani et al., 2016). ARDS commonly exists in intensive care unit (ICU) patients. Despite advanced treatment technologies in ICU, ARDS causes multiple organ dysfunction and high mortality (approximately 30%–45%) (Chiumello et al., 2017). All patients with ARDS undergo ALI, but not all patients with ALI suffer ARDS. The difference between ALI and ARDS lies in the degree of hypoxemia that can be measured by a ratio of arterial partial pressure of oxygen (PaO_2_) and fraction of inspired oxygen (FiO_2_). The PaO_2_/FiO_2_ ratio in ALI is more than 200 mmHg and less than 300 mmHg (regardless of positive end-expiratory pressure (PEEP) level). The PaO_2_/FiO_2_ ratio in ARDS is less than 200 mmHg (regardless of PEEP level) (Bernard et al., 1994). Exosomes (30–150 nm in diameter) represent a class of extracellular vesicles and can be isolated from semen, blood, and breast milk. Exosomes restrain the degradation of cellular molecules and carry cargos (e.g. DNA, RNA, and functional proteins) from donor cells to recipient cells (Kalluri and LeBleu, 2020). Both MALAT1 and miR-425 can be found in plasma exosomes isolated from ARDS patients. MALAT1 hinders the release of miR-425 outside the cells. MALAT1 sponges miR-425 to elevate phosphatase and tensin homolog (PTEN) expression (Wang L. et al., 2019). Plasma exosomes from ARDS patients up-regulate PTEN expression to accelerate cell apoptosis and decrease cell proliferation in A549 and HFL-1 cells (human lung fibroblasts) (Wang L. et al., 2019). He et al. revealed that attenuating endothelial expression of intercellular adhesion molecule-1 (ICAM-1) ameliorates lung inflammation in ARDS mice (He et al., 2018). MALAT1 is highly expressed and miR-150-5p is under-expressed in peripheral blood samples of ARDS patients. MiR-150-5p mimic stimulates miR-150-5p expression, resulting in restraining cell apoptosis rate and the levels of inflammatory cytokines (IL-6, IL-1β, and TNF-α) and adhesion factors (E-selectin and ICAM-1) (Yao et al., 2020). MALAT1 binds to miR-150-5p and inhibits its expression. MALAT1 elimination decreases ICAM-1 expression *via* increasing miR-150-5p, which can hinder cell apoptosis of human PMECs (Yao et al., 2020). Besides, sh-MALAT1 injection decreases lung injury score, lessens total protein and albumin in BALF, and reduces the number of neutrophils and macrophages in BALF. These results suggest that sh-MALAT1 injection relieves lung injury in ARDS mice (Yao et al., 2020).

Taken together, MALAT1 expression is dysregulated during ALI. MALAT1 can positively or negatively regulate ALI (Table 1). MALAT1 mainly modulates cell apoptosis and inflammatory responses in ALI.

**TABLE 1:** Role of lncRNA MALAT1 in acute lung injury.

Models	Expression	Functions	Role	References
ALI	Up	MALAT1↓→miR-181a-5p↑→Fas↓→ cell apoptosis↓, inflammatory responses↓	Promoter	Liu et al. (2021)
ALI	Up	MALAT1↓→miR-194-5p↑→FOXP2↓→cell apoptosis↓	Promoter	Nan et al. (2020)
ALI	Up	MALAT1↓→miR-17-5p↑→FOXA1↓→cell proliferation↑, cell apoptosis↓	Promoter	Wei et al. (2019b)
ALI	Up	MALAT1↓→miR-149↑→MyD88↓→NF-κB↓→inflammatory responses↓	Promoter	Liang et al. (2019)
ALI	Up	MALAT1↓→miR-146a↑→inflammatory responses↓	Promoter	Dai et al. (2018a)
ALI	Down	BML-111→MALAT1↑→TLR4↓→NF-κB↓, p38 MAPK↓→cell apoptosis↓, inflammation↓	Suppressor	Li et al. (2018a)
ARDS	Up	MALAT1↓→miR-150-5p↑→ICAM-1↓→cell apoptosis↓, lung injury↓	Promoter	Yao et al. (2020)

Abbreviations: ALI, acute lung injury; ARDS, acute respiratory distress syndrome; FOXA1, forkhead box protein A1; FOXP2, forkhead box P2; ICAM-1, intercellular adhesion molecule-1.MALAT1, metastasis-associated lung adenocarcinoma transcript 1; MAPK, mitogen-activated protein kinase; miRNA, microRNA; MyD88, myeloid differentiation factor 88; NF‐κB, nuclear factor‐kappaB; TLR4, Toll-like receptor 4.

## LncRNA metastasis-associated lung adenocarcinoma transcript 1 and chronic lung disease

### Chronic obstructive pulmonary disease

COPD is a progressive inflammatory disease with high morbidity and mortality around the world. COPD is mainly induced by tobacco smoking, biomass fuel exposure, and inhalation of smoke from ambient particulate matter (Postma et al., 2015). COPD pathophysiology is characterized by AEC injury, airway remodeling, damage to pulmonary microvasculature, and impaired immune regulation. Patients with COPD often undergo other diseases, like cardiovascular diseases, osteoporosis, muscle weakness, and lung cancer (Rabe and Watz, 2017). Exacerbations of respiratory symptoms in COPD patients are usually triggered by recurrent viral or bacterial infections. The current treatments for COPD include smoking cessation, vaccination (influenza and pneumococcal vaccination), appropriate physical activity, pulmonary rehabilitation, and pharmacologic therapy (Vestbo et al., 2013). Transforming growth factor-β (TGF-β) is a profibrotic cytokine that can stimulate fibronectin expression and α-smooth muscle actin (α-SMA) expression. Treatment of lung fibroblasts (e.g., HFL-1 cell lines) or lung epithelial cells (e.g. BEAS-2B cell lines) with TGF-β can be used as cell models to explore COPD pathogenesis *in vitro* (Tang et al., 2016). LncRNA MALAT1 is over-expressed in lung tissues obtained from COPD patients. In TGF-β-treated HFL-1 cells, repressing MALAT1 boosts cell proliferation and suppresses the expression of fibronectin and α-SMA (Hu et al., 2020). Another study revealed that over-expressed MALAT1 elevates cyclooxygenase two expression *via* inhibiting miR-146a, which can affect pulmonary functions in COPD patients (Sun et al., 2021). Liu et al. indicated that high expression of MALAT1 can be served as a biomarker to predict the acute exacerbation risk and disease conditions in COPD patients (Liu et al., 2020).

### Bronchopulmonary dysplasia

BPD is a neonatal chronic respiratory disease with alveolar simplification and decreased vascular maturation in preterm infants (Simpson et al., 2015). BPD risk factors include genetic susceptibility, pregnancy-induced hypertensive disorders, oxidative stress, hyperoxia, mechanical ventilation, and sepsis (Kalikkot Thekkeveedu et al., 2017). BPD infants usually suffer ineffective gas exchange. Supplemental oxygen therapy and positive airway pressure help BPD infants get respiratory support (Baker and Alvira, 2014). LncRNA MALAT1 expression is elevated in serum samples in BPD newborns compared with that in normal newborns. MALAT1 levels are higher in severe BPD infants than in non-severe BPD infants. MiR-206 is under-expressed in lung tissues in BPD mice and miR-206 up-regulation impairs disease progression in BPD mice (Zhang L. et al., 2021). MALAT1 siRNA can silence MALAT1 expression. MALAT1 siRNA raises miR-206 expression to attenuate lung inflammation, oxidative stress, and cell apoptosis in BPD mice. And MALAT1 knock-down with siRNA increases the number of alveoli and decreases alveolar space and fusion in BPD lungs (Zhang L. et al., 2021). Moreover, MALAT1 can be also served as a biomarker of neonatal BPD. Besides, MALAT1 also has the potential to screen and predict the disease condition of BPD newborns (Zhang L. et al., 2021). High-mobility group box 1 (HMGB1) is a protein associated with inflammatory responses in various respiratory diseases and increased HMGB1 aggravates lung injury (Ding et al., 2017; Zhou M. et al., 2021). MiR-129-5p inhibition improves HMGB1 expression. MALAT1 pcDNA3.1 significantly upregulates MALAT1 expression. MALAT1 pcDNA3.1 promotes cell viability and cell migration in lung epithelial cells (Yangi et al., 2020). Moreover, over-expressed MALAT1 abrogates miR-129-5p to enhance HMGB1 expression, which can contribute to inflammation during BPD development (Yangi et al., 2020). The stimulator of interferon genes (STING) is a transmembrane protein related to lung inflammation in various lung diseases (Benmerzoug et al., 2019). Cyclic adenosine monophosphate (cAMP) response element-binding protein (CREB) is a transcription factor that mediates cellular gene expression (Bartolotti et al., 2016). CREB binds to the STING promoter and improves STING expression. MALAT1 levels are increased in lung tissues collected from the hyperoxia-based rat BPD model. MALAT1 silence decreases CREB phosphorylation and impedes the interaction between CREB and the STING promoter, contributing to attenuating STING expression (Chen J. H. et al., 2020). Ultimately, MALAT1 knockdown stimulates cell proliferation and inhibits cell apoptosis in hyperoxia-treated A549 and BEAS-2B cells. These results reveal a positive relationship between MALAT1 and BPD development (Chen J. H. et al., 2020). Another study reported that suppressing MALAT1 accelerates cell apoptosis in A549 cells exposed to hyperoxia, indicating the protective role of MALAT1 in BPD (Zhang M. et al., 2021). Cai et al. suggested that MALAT1 up-regulation possesses the potential to protect preterm infants with BPD *via* repressing cell apoptosis (Cai et al., 2017).

### Pulmonary fibrosis

Pulmonary fibrosis is a devastating lung disease characterized by progressive and irreversible destruction of lung architecture, resulting in impaired gas exchange and respiratory failure (Wynn, 2011). Bleomycin was used to treat various malignant cancers, but its therapeutic applications are restricted by the accompanied pulmonary toxicity (e.g. pulmonary fibrosis) (Hay et al., 1991). Recently, mice with bleomycin treatment are often used to explore the disease progression of pulmonary fibrosis (Fabre et al., 2008). Macrophages are crucial regulators of pulmonary fibrosis. Macrophages in the lungs are divided into alveolar macrophages and interstitial macrophages. In response to different environmental stimuli, macrophages exhibit two distinct polarization states: M1 phenotype and M2 phenotype. M1 macrophages secrete pro-inflammatory cytokines, like TNF-α, IL-1β, and IL-6. M2 macrophages generate anti-inflammatory cytokines, including IL-4, IL-10, and IL-13 (Laskin et al., 2019). LncRNA MALAT1 expression is up-regulated in LPS-treated macrophages. MALAT1 deficiency diminishes C-type lectin domain family 16, member A (Clec16a) to repress the pro-inflammatory polarization of macrophages. MALAT1 knockout decreases BALF inflammatory cytokine (TNF-α, IL-1β, and IL-6) levels, hinders lung neutrophil infiltration, and impedes lung interstitial thickening and deposition of the hyaline membrane. MALAT1 deficiency ultimately relieves LPS-induced ALI in mice (Cui et al., 2019). While MALAT1 expression is decreased in resident alveolar macrophages isolated from fibrotic lungs. MALAT1 deletion promotes glucose-derived mitochondrial oxidative phosphorylation (OxPhos) to boost both M2 polarization and profibrotic differentiation of macrophages, which leads to the progression of bleomycin-induced pulmonary fibrosis in mice (Cui et al., 2019). Epithelial-mesenchymal transition (EMT) is a process that epithelial cells lose cell-cell contacts and transform into mesenchymal cells. The EMT can be regulated by several transcription factors, including Snail, Zeb, and Twist. And EMT plays an essential role in pulmonary fibrosis (Nieto et al., 2016). MALAT1 expression is over-expressed in silica-treated human bronchial epithelial cells. MiR-503 expression is declined in the lung tissues collected from mice with silica-induced lung fibrosis. MiR-503 targets and suppresses phosphatidylinositol-3-kinase (PI3K) p85, leading to impairing protein kinase B (AKT)/mammalian target of rapamycin (mTOR)/Snail signaling pathway (Yan et al., 2017). MALAT1 binds to miR-503 and its down-regulation increases miR-503 expression. Eventually, MALAT1 deficiency abrogates EMT *via* targeting the miR-503-mediated PI3K/AKT/mTOR/Snail pathway, which can repress silica-induced pulmonary fibrosis (Yan et al., 2017).

### Asthma

Asthma is an allergic airway disorder affecting an estimated 334 million people in the world (Vos et al., 2012). Asthma symptoms include recurrent wheezing, chest tightness, cough, as well as shortness of breath. Asthma is characterized by reversible airway narrowing and airway hyperresponsiveness caused by nonspecific stimuli (e.g. air pollutants and allergens). The main contributors to asthma include airway inflammation and airway remodeling (Russell and Brightling, 2017). Airway smooth muscle cells (ASMCs), the main structural component in the airway, affect airway inflammation *via* the secretion of various cytokines, chemokines, and growth factors (Zuyderduyn et al., 2008). ASMC mass is one of the strongest predictors of airflow obstruction. And abnormal ASMC migration and proliferation are responsible for airway remodeling in asthma (Brightling et al., 2012; Salter et al., 2017). MiR-133a expression is decreased in lung sections isolated from asthmatic mice. Over-expressed miR-133a reduces airway remodeling in asthma (Shao et al., 2019). Ryanodine receptor 2 (RyR2) is a risk gene factor for childhood allergic asthma. RyR2 is a targeted gene of miR-133a (Yang and Wang, 2021). LncRNA MALAT1 expression is increased in tracheal tissues of asthma modeling rats. MALAT1 knockdown decreases RyR2 expression and augments miR-133a levels in bronchial smooth muscle cells (BSMCs). Down-regulated MALAT1 represses cell apoptosis and inflammatory factor (IL-6, TNF-α, and IL-1β) release through the miR-133a-RyR2 axis in BSMCs (Yang and Wang, 2021). MALAT1 is upregulated and miR-216a is down-regulated in serum samples of asthmatic patients. Inhibiting MALAT1 increases miR-216a to facilitate cell apoptosis and suppress proliferation and migration in asthma AMSCs (Huang et al., 2021). Platelet-derived growth factor-BB (PDGF-BB) can induce AMSC proliferation and migration *in vitro* (Dai Y. et al., 2018). MiR-150 expression is declined in PDGF-BB-treated AMSCs. MiR-150 interacts with 3′UTR of eukaryotic initiation factor 4E (eIF-4E). Both over-expressed miR-150 and eIF-4E suppression reduce the expression of phosphorylated AKT (Lin L. et al., 2019). MALAT1 abrogates miR-150 expression and induces eIF-4E expression. Suppressing MALAT1 impairs PDGF-BB-stimulated ASMC proliferation and migration *via* increasing miR-150 and blocking eIF4E/AKT signaling (Lin L. et al., 2019). It is believed that CD4^+^ T cells can be divided into two distinct cell subsets: T helper 1 (Th1) cells and Th2 cells. CD4^+^ T cells with a Th2-cytokine profile participate in allergic inflammation in asthma (Mazzarella et al., 2000). Increased Th2/Th1 ratio exacerbates asthma (Gu et al., 2022). The transcription factors T box protein expressed in T cells (T-bet) and GATA-binding protein 3 (GATA-3) can facilitate the expression of Th1-type cytokines (Szabo et al., 2000) and Th2-type cytokines (Fields et al., 2002), respectively. Th2 cytokines (IL-4, IL-5, and IL-13) promote eosinophilic inflammation and then contribute to airway constriction and remodeling in asthma (Foronjy, 2020). MiR-155 is down-regulated in asthmatic patients. MiR-155 inhibits cytotoxic T-lymphocyte antigen 4 (CTLA4). Both MALAT1 down-regulation and miR-155 mimic elevate the levels of Th1-type cytokines (e.g. IFN-γ, and IL-2) and T-bet in CD4^+^ T cells (Liang and Tang, 2020). Moreover, both MALAT1 up-regulation and miR-155 inhibitor promote the expression of Th2-type cytokines (e.g., IL-4, and IL-10) and GATA3 in CD4^+^ T cells. As a result, increased MALAT1 abrogates miR-155 to cut down the Th1/Th2 ratio and T-bet/GATA3 ratio within CD4^+^ T cells *via* CTLA4 (Liang and Tang, 2020). Liao et al. suggested that MALAT1 can be served as a potential therapeutic target and prognostic biomarker in asthma (Liao et al., 2020).

### Pulmonary hypertension

PH has been defined hemodynamically by a resting mean pulmonary arterial pressure of higher than 20 mm Hg, as measured by right heart catheterization (Simonneau et al., 2019). PH is divided into five principal forms: a) PAH, b) PH due to left-sided heart disease (e.g., left-sided valvular diseases, cardiomyopathies), c) PH related to lung disease, hypoxia, or both, d) PH related to pulmonary-artery obstructions, and e) PH with multifactorial or unclear mechanisms (Hoeper et al., 2017; Hassoun, 2021). The remodeling of distal pulmonary vasculature is one of the common pathological features of PAH, which is associated with abnormal fibroblast proliferation, endothelial cell apoptosis and proliferation, smooth-muscle cell hyperplasia, inflammatory cell recruitment, and collagen disruption. These changes during PAH contribute to luminal narrowing or complete occlusion of small vessels (Hassoun, 2021). PAH symptoms are nonspecific, including dyspnea, fatigue, chest pain, fluid retention, and near-syncope. The endothelin-receptor antagonists, nitric oxide, phosphodiesterase type 5 inhibitors, and prostacyclin derivatives can be used to treat PAH (Humbert et al., 2004). LncRNA MALAT1 is upregulated in endothelial cells in response to hypoxia. MALAT1 small interfering RNA (siRNA) significantly diminishes MALAT1 levels. MALAT1 siRNA treatment represses endothelial cell proliferation, which can impair vascularization (Michalik et al., 2014). One study found that miR-503 expression is declined in lungs collected from PH rats. And over-expression of miR-503 alleviates experimental PH *in vivo* (Kim et al., 2013). MiR-503 targets TLR4. MALAT1 is highly expressed in plasma in PAH patients. MALAT1 sponges miR-503 to enhance TLR4 expression. Functionally, in human pulmonary artery smooth muscle cells (PASMCs), MALAT1 reduces cell apoptosis and promotes cell proliferation and migration through the miR-503-TLR4 signal axis. MALAT1 can serve as a potential biomarker for PAH diagnosis (He et al., 2020). Kruppel-like factor 5 (KLF5), a transcription factor, has been proven to regulate vascular remodeling through hypoxia-inducible factor 1α (HIF1α) in hypoxic PH (Li et al., 2016). MALAT1 acts as a ceRNA for miR-124-3p.1 that targets KLF5. Down-regulated MALAT1 increases miR-124-3p.1 expression to abrogate KLF5 expression, leading to attenuating cell proliferation and migration in human PASMCs (Wang D. et al., 2019). MALAT1 expression can be elevated by hypoxia *via* HIF1α in hypoxia-treated human PASMCs. On the one hand, MALAT1 depletion inhibits cell migration *in vitro* (Brock et al., 2017). On the other hand, MALAT1 silence increases the mRNA levels of CDK inhibitors (e.g. CDKN1B, CDKN1C, CDKN2A, CDKN2B, CDKN2C, and CDKN2D), resulting in suppressing cell proliferation in human PASMCs. In addition, knocking out MALAT1 also reduces heart hypertrophy in mice with hypoxia-induced PH (Brock et al., 2017). It has been shown that MALAT1 genetic polymorphisms are correlated with disease susceptibility (Gong et al., 2016). Endothelial cells transfected with pcDNA-*MALAT1*-rs619586G show lower proliferation and migration capabilities than those cells transfected with pcDNA-*MALAT1*-rs619586A. The rs619586A > G (allele alteration from A to G) single nucleotide polymorphism in the *MALAT1* gene possesses the potential to indicate decreased PAH susceptibility (Zhuo et al., 2017).

## Lncmetastasis-associated lung adenocarcinoma transcript 1 and other conditions

Lung transplantation is a life-saving therapeutic option for patients with end-stage respiratory diseases. Ischemia-reperfusion injury (IRI) after lung transplantation is a severe complication and contributes to primary graft dysfunction (Chen-Yoshikawa, 2021). The p300 histone acetyltransferase is a transcriptional coactivator and involves in cell proliferation, cell apoptosis, and DNA damage response (Banerjee et al., 2012). The p300 over-expression promotes IL-8 expression *via* H3K27 acetylation (H3K27ac) enrichment. LncRNA MALAT1 expression is increased in lung tissues collected from rats undergoing lung transplant ischemia-reperfusion (LTIR). MALAT1 elevates IL-8 transcription *via* recruiting p300 (Wei L. et al., 2019). MALAT1 deficiency abrogates infiltration and activation of neutrophils *via* the p300-regulated downregulation of IL-8 *in vivo*. Besides, MALAT1 deletion also decreases the lung drying wet ratio. As a result, knocking out MALAT1 ameliorates inflammatory injury related to LTIR in rats (Wei L. et al., 2019). Previous studies have discovered that upregulation of adhesion molecules contributes to radiation-induced lung injury in mice (Hallahan et al., 2002). MALAT1 expression can be raised by irradiation-induced DNA damage through cGAS (a DNA sensor). Under irradiation exposure, MALAT1 enhances the expression of signal transducer and activator of transcription 1 (STAT1) *via* sponging miR-146a, which can facilitate the expression of adhesion molecules, including ICAM-1 and E-selectin. Thereby, MALAT1 promotes irradiation-induced inflammatory response and lung injury in mice lung (Yuan et al., 2020). MALAT1 expression is higher in sepsis blood samples compared with normal blood samples. Increased MALAT1 boosts cell apoptosis of human bronchial epithelial cells in response to LPS (Yue et al., 2022). Pneumonia is one of the commonest respiratory infections that can damage lung alveoli and distal airways. Pneumonia occurs in all ages and can be caused by bacteria (e.g., *Mycoplasma pneumoniae* (MP), and *Legionella pneumophila*), respiratory viruses, and fungi (Torres et al., 2021). MALAT1 expression is up-regulated in BALF obtained from children with MP pneumonia. Knocking out MALAT1 represses NF-κB activation to alleviate lung inflammation and lung injury induced by MP infection. MALAT1 suppression also inhibits cell infiltration, lessens alveolar wall thickening, decreases secretion, and blocks structural collapse in lung tissues in mice with MP infection (Gu et al., 2020).

## Targeted therapy

As mentioned above, lncRNA MALAT1 involves in various cellular processes of lung injury, suggesting its potential to act as a therapeutic target. Phytochemicals paclitaxel and resveratrol are used to treat human diseases. Paclitaxel can restrain sepsis-mediated ALI *via* attenuating the TLR-4/NF-κB pathway (Wang Y. M. et al., 2019). And paclitaxel inhibits MALAT1 and then increases miR-370-3p expression, leading to repressing HMGB1 expression. Paclitaxel eventually alleviates LPS-induced acute kidney injury (Xu et al., 2020). Resveratrol decreases permeability as well as reduces apoptosis of alveolar epithelium in methamphetamine-regulated chronic lung injury (Wang X. et al., 2020). MALAT1 targets and declines miR-22-3p. The NOD-like receptor family pyrin domain containing 3 (NLRP3) is a downstream target of miR-22-3p. Resveratrol blocks pulmonary embolism-caused cardiac injury by impairing the MALAT1-miR-22-3p-NLRP3 axis (Yang et al., 2019). Taken together, some therapies can target MALAT1 to relieve tissue injury.

### Minocycline

Minocycline is a lipophilic semi-synthetic tetracycline with anti-bacterial and anti-inflammatory properties. Minocycline shows efficacy in experimental models of various diseases, including ischemia, neuropathic pain, and brain injury (Garrido-Mesa et al., 2013). Cui et al. found that minocycline blocks oxidative stress and inflammatory responses *via* suppressing MALAT1, contributing to ameliorating septic lung injury in ALI mice (Cui et al., 2021).

### Propofol

Propofol is an intravenous anesthetic widely used in operating rooms. Propofol possesses neuroprotective and anti-inflammatory effects. It can diminish cerebral blood flow and decrease intracranial pressure (Kotani et al., 2008). Autophagy, a catabolic process, controls human body homeostasis and is correlated with varied disorders, like cancer and neurodegenerative diseases (Saha et al., 2018). MALAT1 abrogates miR-144 which restricts the expression of glycogen synthase kinase-3β (GSK3β). MALAT1 over-expression stimulates cell apoptosis and increases lung drying wet ratio and lung injury score in lung tissues of IRI mice (Zhang J. P. et al., 2021). Propofol impedes autophagy activation and secretion of inflammatory factors (e.g., TNF-α, IL-1β, and IL-18) *via* impairing the MALAT1-miR-144-GSK3β signaling axis *in vitro* and *in vivo*. Propofol eventually relieves lung IRI in mice with ischemia-reperfusion (Zhang J. P. et al., 2021).

### Dexmedetomidine

Dexmedetomidine (DEX), an adrenergic receptor agonist, is commonly used in ICU and operating rooms due to its good sedative effects (Shi et al., 2021). Increasing evidence determined that endoplasmic reticulum stress (ERS) activates unfolded protein response (UPR) to give rise to tissue injury (Chen X. et al., 2019). X-box binding protein-1 (XBP-1) is an essential regulator of UPR. XBP-1 includes active isoform (XBP-1S) and inactive isoform (XBP-1 U). One study demonstrated that decreased ratio of XBP-1S/XBP-1U results in suppressing ERS (Ge et al., 2021). MALAT1 over-expression abates miR-135a-5p to attenuate XBP-1S and elevated XBP-1 U. As a result, MALAT1 up-regulation suppresses ERS, ultimately leading to inhibiting inflammation responses and cell apoptosis in ALI rats (Li et al., 2021). DEX treatment can mitigate pulmonary edema, rupture of the alveolar septum, and the infiltration of inflammatory cells and red blood cells in lung tissues of ALI rats. And the protective effect of DEX on ALI can be strengthened by MALAT1 up-regulation (Li et al., 2021).

## Discussion and conclusion

Both acute and chronic lung diseases impair the normal lung microenvironment, which can lead to lung injury. Lung injury is recognized as an inflammatory process demonstrated as the disability of lung capillary endothelial and alveolar epithelial cells. Lung injury results from the release of inflammatory cytokines (e.g., IL-6, IL-1, and TNF-α) caused by the activation of inflammatory immune cells (e.g., monocytes and macrophages) in the pulmonary luminal epithelium (Liu et al., 2019). Lung injury is mostly associated with lung inflammation (Kumar, 2020). Accumulative evidence proved that chronic lung inflammation contributes to lung cancer development (Ballaz and Mulshine, 2003). For example, COPD serves as an independent risk factor for lung cancer (Huang et al., 2015). LncRNA MALAT1 was first found in the early stage of NSCLC. MALAT1 can predict the metastasis and survival of early NSCLC (Ji et al., 2003). MALAT1 has been widely studied in lung cancer. For instance, MALAT1 can accelerate proliferation, migration, and invasion through the miR-185-5p/mouse double minute 4 (MDM4) axis in NSCLC (Wang D. et al., 2020). Increased MALAT1 enhances KLF4 expression *via* repressing miR-145, which can lead to cisplatin resistance in NSCLC (Cui et al., 2018). Besides, MALAT1 can be acted as a biomarker for the early diagnosis of lung cancer (Zhou Q. et al., 2021). There are many studies related to MALAT1 in lung cancer. And the role of MALAT1 in lung cancer progression has been widely explored. However, the role of MALAT1 in acute and chronic lung diseases has not been fully elucidated. In the present review, we try to figure out the relationships between lung injury and MALAT1 by summarizing the evidence we collected. However, a large portion of the mechanisms remains unclear and may require further experimental exploration.

**FIGURE 1:**
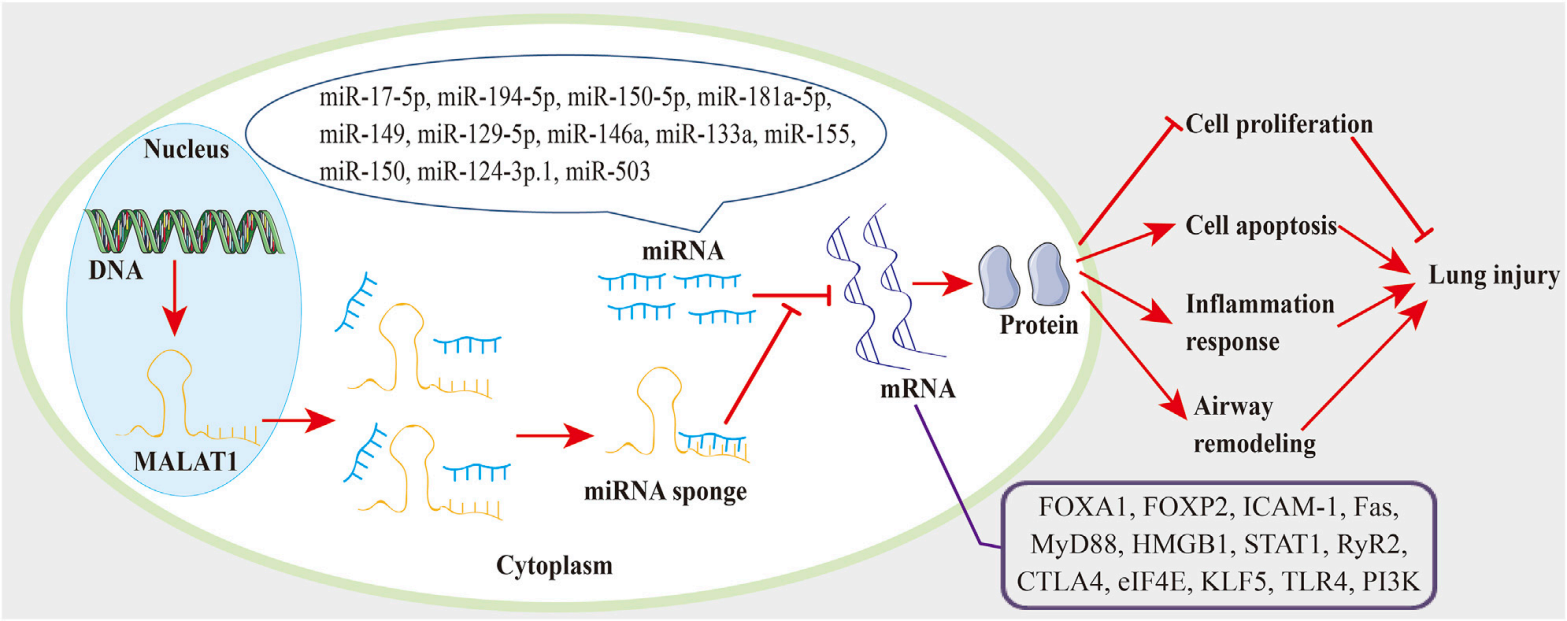
The MALAT1-miRNA-mRNA regulatory network in lung injury. MALAT1 acts as a miRNA sponge to reduce miRNA activity and abrogate miRNA inhibition on targeted mRNAs. Thereby, MALAT1 regulates cell proliferation, cell apoptosis, inflammation response, and airway remodeling through the miRNA-mRNA axis, which can eventually affect the progression of lung injury.

In the current review, we discussed how MALAT1 can exert positive or negative roles in ALI (Table 1) and chronic lung diseases (Table 2). Decreased cell proliferation, increased cell apoptosis, and enhanced inflammatory cytokine release promote ALI. During ALI, a) MALAT1 positively regulates cell apoptosis through the miR-181a-5p-Fas axis (Liu et al., 2021), miR-194-5-FOXP2 axis (Nan et al., 2020), miR-17-5p-FOXA1 axis (Wei S. et al., 2019), and miR-150-5p-ICAM-1 axis (Yao et al., 2020); b) MALAT1 positively regulates inflammatory responses *via* the miR-181a-5p-Fas axis (Liu et al., 2021) and miR-149-MyD88-NF-κB axis (Liang et al., 2019); c) MALAT1 also exhibits negative effects on cell apoptosis and lung inflammation through the TLR4-NF-κB-p38 MAPK axis (Li H. et al., 2018). In COPD, MALAT1 negatively regulates cell proliferation (Hu et al., 2020). In BPD, a) MALAT1 positively regulates lung inflammation *via* miR-206 (Zhang L. et al., 2021) and miR-129-5p-HMGB1 axis (Yangi et al., 2020); b) MALAT1 positively regulates cell apoptosis *via* miR-206 (Zhang L. et al., 2021) and CREB-STING axis (Chen J. H. et al., 2020); c) MALAT1 also shows inhibitory roles in cell proliferation (Chen J. H. et al., 2020) and cell apoptosis (Cai et al., 2017; Zhang M. et al., 2021). In pulmonary fibrosis, a) MALAT1 positively regulates EMT *via* miR-503/PI3K/AKT/mTOR/Snail pathway (Cui et al., 2019); b) MALAT1 inhibits M2 macrophage polarization and profibrotic differentiation of macrophage *via* OxPhos (Cui et al., 2019). In asthma, MALAT1 improves cell proliferation and cell migration through miR-216a (Huang et al., 2021) and miR-150-eIF4E-AKT (Lin L. et al., 2019). In PH, MALAT1 positively regulates cell proliferation and cell migration *via* the miR-503-TLR4 axis (He et al., 2020) and miR-124-3p.1-KLF5 axis (Wang D. et al., 2019). In LTIR, MALAT1 promotes infiltration and activation of neutrophils *via* IL-8 (Wei L. et al., 2019). In radiation-induced lung injury, MALAT1 promotes inflammatory response and lung injury through the miR146a-STAT1 axis (Yuan et al., 2020). In pneumonia, MALAT1 positively regulates lung inflammation and lung injury *via* NF-κB (Gu et al., 2020). In this review, we also found that the lncRNA-miRNA-mRNA ceRNA regulatory network (Figure 1) participates in lung injury. And this ceRNA network exists in ALI (Wei S. et al., 2019; Liang et al., 2019; Nan et al., 2020; Yao et al., 2020; Liu et al., 2021), BPD (Yangi et al., 2020), pulmonary fibrosis (Yan et al., 2017), asthma (Lin L. et al., 2019; Liang and Tang, 2020; Yang and Wang, 2021), PH (Wang D. et al., 2019; He et al., 2020), and radiation-induced lung injury (Yuan et al., 2020). And some therapies can impair the lncRNA-miRNA-mRNA axis to inhibit lung injury. For instance, propofol blocks the MALAT1-miR-144-GSK3β signaling axis to suppress lung IRI (Zhang J. P. et al., 2021). These results suggest a promising treatment strategy based on the lncRNA-miRNA-mRNA axis for lung injury. In the present review, we also found that minocycline (Cui et al., 2021) and DEX (Li et al., 2021) can target MALAT to alleviate lung injury. These suggest that therapies involving MALAT1 may have potential to treat lung injury.

**TABLE 2:** Role of lncRNA MALAT1 in chronic lung diseases and other conditions.

Lung diseases	Expression	Functions	Role	References
COPD	Up	MALAT1↓→cell proliferation↑	Promoter	Hu et al. (2020)
BPD	Up	MALAT1↓→miR-206↑→lung inflammation↓, oxidative stress↓, cell apoptosis↓	Promoter	Zhang et al. (2021b)
	Up	MALAT1↑→miR-129-5p↓→HMGB1↑→inflammation↑	Promoter	Yangi et al. (2020)
	Up	MALAT1↓→CREB phosphorylation↓→STING↓→cell proliferation↑, cell apoptosis↓	Promoter	Chen et al. (2020a)
	Down	MALAT1↓→cell apoptosis↑	Suppressor	Zhang et al. (2021c)
	Up	MALAT1↑→cell apoptosis↓	Suppressor	Cai et al. (2017)
Pulmonary fibrosis	Down	MALAT1↓→OxPhos↑→M2 macrophage polarization↑, profibrotic differentiation of macrophage↑→pulmonary fibrosis↑	Suppressor	Cui et al. (2019)
	Up	MALAT1↓→miR-503↑→PI3K/AKT/mTOR/Snail pathway↓→EMT↓→pulmonary fibrosis↓	Promoter	Yan et al. (2017)
Asthma	Up	MALAT1↓→miR-133a↑→RyR2↓→cell apoptosis↓, inflammatory factor release↓	Promoter	Yang and Wang, (2021)
	Up	MALAT1↓→miR-216a↑→cell apoptosis↑, proliferation↓, migration↓	Promoter	Huang et al. (2021)
	Up	MALAT1↓→miR-150↑→eIF4E↓→AKT↓→proliferation↓, migration↓	Promoter	Lin et al. (2019a)
	Up	MALAT1↑→miR-155↓→CTLA4↑→Th1/Th2 ratio↓, T-bet/GATA3 ratio↓	Promoter	Liang and Tang, (2020)
PH	Up	MALAT1↑→miR-503-TLR4 signal axis↓→cell apoptosis↓, cell proliferation↑, migration↑	Promoter	He et al. (2020)
	Up	MALAT1↓→miR-124-3p.1↑→KLF5↓→proliferation↓, migration↓	Promoter	Wang et al. (2019a)
	Up	MALAT1↓→proliferation↓, migration↓	Promoter	Brock et al. (2017)
LTIR	Up	MALAT1↓→IL-8↓→infiltration and activation of neutrophils↓→inflammatory injury↓	Promoter	Wei et al. (2019a)
Radiation-induced lung injury	Up	Irradiation→DNA damage↑→MALAT1↑→miR146a↓→STAT1↑→adhesion molecules↑→inflammatory response↑, lung injury↑	Promoter	Yuan et al. (2020)
Pneumonia	Up	MALAT1↓→NF-κB↓→lung inflammation↓, lung injury↓	Promoter	Gu et al. (2020)

Abbreviations: COPD, chronic obstructive pulmonary disease; MALAT1, metastasis-associated lung adenocarcinoma transcript 1; miRNA, microRNA; BPD, bronchopulmonary dysplasia; HMGB1, high-mobility group box 1; CREB, cyclic adenosine monophosphate (cAMP) response element-binding protein; STING, stimulator of interferon genes; OxPhos, oxidative phosphorylation; PI3K, phosphatidylinositol-3-kinase; AKT, protein kinase B; mTOR, mammalian target of rapamycin; EMT, epithelial-mesenchymal transition; RyR2, ryanodine receptor 2; eIF-4E, eukaryotic initiation factor 4E; CTLA4, cytotoxic T-lymphocyte antigen 4; Th1, T helper one; T-bet, T box protein expressed in T cells; GATA3, GATA-binding protein 3; PH, pulmonary hypertension; TLR4, Toll-Like Receptor 4; KLF5, Kruppel-like factor 5; LTIR, lung transplant ischemia-reperfusion; IL-8, interleukin-8; STAT1, signal transducer and activator of transcription 1; NF-κB, nuclear factor‐kappaB.

Recently, increasing evidence reveals that inhalation-based delivery of siRNAs is a potential strategy for the treatment of lung injury (Zoulikha et al., 2022). Patisiran is a nanoparticle formulation that contains a chemically modified siRNA encapsulated in a lipid nanoparticle. Patisiran is the first siRNA therapeutic agent approved for the treatment of hereditary transthyretin-mediated amyloidosis in adults (Hoy, 2018). The clinical practice of the first siRNA treatments provides new sights for further exploration of this therapeutic strategy for other diseases. MALAT1 shRNA and siRNA significantly reduce MALAT1 expression. Intravenous injection of MALAT1 shRNA relieves lung injury in ALI rats (Dai L. et al., 2018). Subcutaneous injection of MALAT1 siRNA suppresses lung injury in the BPD rat model (Zhang L. et al., 2021). These suggest the potential of MALAT1 siRNA/shRNA in the treatment of lung injury.

Presently, despite the great progress that has been made in lung injury, researches based on the MALAT1-miRNA-mRNA axis are still lacking. It is widely suggested that LPS-induced inflammation can mimic the microenvironment in lung injury. Some other agents can also induce animal models of lung injury, such as oleic acid, acid aspiration, and mechanical ventilation (Matute-Bello et al., 2008). The animal models may be useful to explore the pathological mechanism of lung injury.
